# Alternative Strategy for Alzheimer's Disease: Stress Response Triggers

**DOI:** 10.1155/2012/684283

**Published:** 2012-05-10

**Authors:** Joan Smith Sonneborn

**Affiliations:** Department of Zoology and Physiology, University of Wyoming, Laramie, WY 82071, USA

## Abstract

Stress resistance capacity is a hallmark of longevity protection and survival throughout the plant and animal kingdoms. Latent pathway activation of protective cascades, triggered by environmental challenges to tolerate heat, oxygen deprivation, reactive oxygen species (ROS), diet restriction, and exercise provides tolerance to these stresses. Age-related changes and disease vulnerability mark an increase in damage, like damage induced by environmental challenges. An alternative approach to immunotherapy intervention in Alzheimer's Disease is the use of mimetics of stress to upregulate endogenous protective cascades to repair age damage, shift the balance of apoptosis to regeneration to promote delay of onset, and even progression of Alzheimer's disease memory dysfunction. Mimetics of environmental stress, hormetic agents, and triggers, endogenous or engineered, can “trick” activation of expression patterns of repair and rejuvenation. Examples of known candidate triggers of heat response, endogenous antioxidants, DNA repair, exercise, hibernation, and telomeres are available for AD intervention trials. Telomeres and telomerase emerge as major regulators in crossroads of senescence, cancer, and rejuvenation responsive to mimetics of telomeres. Lessons emerge from transgenic rodent models, the long-lived mole rat, clinical studies, and conserved innate pathways of stress resistance. Cross-reaction of benefits of different triggers promises intervention into seemingly otherwise unrelated diseases.

## 1. Introduction

 Divergent biological phenomena have fundamental convergent pathways that affect aging, age-related diseases, and stress resistance responses. Hormetic stress pathways are activated by environmental chemical and physical cues, that are beneficial at threshold low levels but are otherwise toxic agents at higher levels [1]. Nature preserves those organisms and small molecular triggers that promote tolerance responses to environmental stress including, youthful restoration of DNA repair, resistance to oxidizing agents, protein structure and function repair, improved immunity, tissue remodeling, and altered metabolism [2]. Survival pathways in ancient species exist in present species and when activated, show potential for increased longevity and latent rejuvenation potential regardless of divergence of the hormetic stressing agent. Environmental stress of UV and photoreaction, activates survival pathways to rejuvenate cells and increase lifespan in paramecia [3], and induces radiation resistance and DNA repair in human cells in culture [4]. Common key regulators and pathways respond to diverse challenges of physical and chemical stresses of temperature, diet, exercise, hibernation, and radiation. Both posttranslational and transcriptional activation of latent pathways responses involves epigenetic modifications by deacetylation, phosphorylation, methylation, ubiquitination, and mechanisms used in differentiation to provide stress resistance. As a consequence of common protective pathways, cross-resistance to pathologies that share common cellular cues represents an under-used strategy in disease intervention; that is, drugs effective in divergent diseases may show benefit in acute and chronic dysfunctions and have application in intervention in Alzheimer's disease.

 The goal of intervention strategy reviewed here is to decrease vulnerability and rescue in Alzheimer's disease, by activation of stress resistance pathways. Triggers mimic environmental stresses including oligonucleotides, heat shock, exercise, and hibernation drugs, known to activate key regulators of protective metabolic pathways to restore homeostasis, and proposed to provide resistance and repair of oxidative DNA and protein damage induced by AD.

 This review focus is on lessons learned from the role of stress resistance triggers, hormesis, and telomeres, in rodent models of induced senescence, successful aging in the mole rat, and obstacles encountered in immunological therapy in clinical studies to provide a basis for intervention strategy for AD.

## 2. Mimetics of Stress Resistance

 Stress resistance is key for survival and maintenance of the species, and nature has preserved survival pathways from single cells to man. Since the appropriate threshold of low-dose beneficial versus toxic dose of environmental and chemical stress is difficult to assess, the use of mimetic agents of these stresses offers better dosage control to avoid high-dose stress damage [2]. Mimetics can trigger stress-related transcriptomes, expression of families of genes activated by a common transcription factor, that provide benefit not only the targeted beneficial response, but also youthful rejuvenation, and improvement of multiple avenues to stress resistance to intervene in multiple age-related disease [5–7]. These fundamental survival pathways, lifespan assurance loci, master regulators, also called vitagenes, confer plasticity to species longevity, lifespan extension, rejuvenation, and repair [2, 5–12]. As the molecular roles of aging, stress and neurodegenerative disease are elucidated, oxidative stress emerges as a common damage denominator and activation of pathways used in early development; that is, FOXO and IGF-1, also serve roles in mitigation of stress resistance and disease [13–15].

## 3. Radiation Stress

Mimetics of UV damage include the use of DNA oligonucleotides homologous to the telomere (TTAGGG repeat, “T-oligo's”) as triggers to activate innate telomere-based protective responses that act to reduce DNA and oxidative damage to cells [4]. The antioxidative pathways induction, by T-oligo's, makes these UV mimetics potential candidates for relief from induced oxidative toxicity in AD and cancer. More recently, telomere homolog oligonucleotides show induction of apoptosis in malignant, and not normal lymphoid cells, to provide potential anticancer therapy potential [16].

## 4. Protein Structure and Function Stress Damage

Protein misfolding and aggregation from single cells to multicellular organisms dramatically affect normal cell structure and function needed for survival [17] and is a hallmark of AD. The rescue of neuron protein damage involves activation of the heat shock response, and FOXO, and SIRT-1 to restore protein homeostasis [18, 19]. Protein homeostasis (proteostasis) is achieved by how high the threshold of the stress response is set to detect and combat protein misfolding. The heat shock factor 1 (HSF1) regulates the response to the metabolic state of the cell and centralized neuronal control that allows optimal resource allocation between cells and tissues. HSF1 activation requires a stress-activated NAD+-dependent SIRT1 deacetylase and phosphorylation, to signal transcription of molecular chaperones that resolve misfolded and aggregated proteins [19]. Misfolded proteins, whether a consequence of aging, toxins, hypoxic, oxidative, or ischemic stress, signal cell death damage, proapoptosis responses, that impact longevity, and disease states. HSP 70 heat shock protein is a major rescue response to damage that impacts longevity [20, 21], vulnerability, and progression of AD neuronal pathology. Hormetic agents are candidates to intervene in proteotoxic damage and associated clinical symptoms [22, 23] and are identified here.

 Ethanol is a candidate hormetic trigger to induce the heat shock response [23] and thus has potential for intervention in AD. Ethanol preconditioning inhibits amyloid-Beta-induced neurotoxicity and apoptosis [24]. Constitutive and inducible HSP70s are involved in oxidative resistance evoked by heat shock and ethanol. In the brain, moderate ethanol pretreatment causes an almost 3-fold increase in brain levels of heat shock protein HSP 70 and can prevent beta-amyloid peptide (Abeta)-induced neurotoxicity and apoptosis in organotypic hippocampal-entorhinal slice cultures [24]. Neuronal protection by ethanol pretreatment reduces behavioral deficit, neuronal death, and delays neuronal death, neuronal and dendritic degeneration, oxidative DNA damage, and glial-cell activation after ischemia/reperfusion (I/R) challenge [25] and prevents postischemic leukocyte-endothelial cell-adhesive interactions [26].

 Another trigger of protection against oxidative damage known to induce endogenous antioxidants and HSP70 is an acyclic isoprenoid. Geranylgeranylacetone (GGA) is a nontoxic HSP70 inducer of HSP70 with beneficial responses including reduction of inflammation in gastritis, apoptosis, induction of protective pathways like thioredoxin, and antiviral genes that offer a generalized upregulation of disease immunity [27, 28].

 Activation of endogenous antioxidants is an alternative approach to upregulate natural defenses against oxidative damage and associated pathologies of neurodegenerative disease and including AD. Activators of the “Antioxidant Response Element” include oltipraz, and ferritins. Oltipaz is a substituted 1,2-dithiole-3-thione, originally developed as an antischistosomal agent, that possesses chemopreventive activity by transcriptional activation of a gene cascades involved in carcinogen detoxification and attenuation of oxidative stress [29]. Exposure of rodents to 1,2-dithiole-3-thiones trigger nuclear accumulation of the transcription factor Nrf2 and its enhanced binding to the “antioxidant response element” (ARE).

 Ferritins, an ancient family of protein nanocages, also participate in activation of the ARE-responsive element. Ferritins concentrate iron in iron-oxy minerals for iron-protein biosynthesis and protection against oxy radical damage. The promoter of human ferritin-L contains an overlapping Maf recognition element (MARE) antioxidant responsive element (ARE). Thoreductase can be transcriptionally activated by sulphorane and other electophiles by the antioxidant response element ARE. The ferritin receptor is activated by tert*-*butylhydroquinone, sulforaphane, and hemin with responses comparable to thioredoxin reductase, ARE regulator or quinone reductase (MARE/ARE regulator) [30].

## 5. Hibernation and AD Intervention

 Hibernation is a classical beneficial response to environmental stresses of depleted energy stores, intracellular acidosis, hypoxia, hypothermia, cell volume shifts, and inactivity induced muscle wasting [31] characterized by epigenetic modulation affecting transcriptional and translational controls [32, 33]. Animals do not need to undergo a torpor state, to benefit from activation of at least some of the hibernation protective pathways. Use of Hibernation Induction Triggers, identified to activate protective hibernation gene cascades, especially using deltorphins opioid receptor agonists as mimetics of hibernation, shows reduction of damage in rodent model systems of heart attack [7], stroke, and hemorrhage shock [34–39]. The cardioprotective mechanism of deltorphin II is mediated via stimulation of peripheral delta (2) opioid receptors that involve protein kinase C, NO-synthase, KATP, and the autonomic nervous system to induce both its infarct-sparing and antiarrhythmic effects [37]. Neuroprotection by both hibernating woodcock serum and deltophin E was demonstrated in an neuronal ischemic stress rodent model [38]. The delta-2 opioid receptor agonist activation of protective pathways includes anti-inflammatory properties [39] that likely contribute to proven resistance to shock, that may also reduce AD pathology and progression.

 Metabolic changes also characterize hibernation. Upregulation of fatty acid-binding proteins during hibernation facilitates the switch to a primary dependence on lipid fuels by nearly all organs. Changes in hibernation include upregulation of key regulators of energy metabolism and mitochondrial biogenesis, namely, PPAR gamma transcription factor and its coactivator, PGC-1. Several hypoxia-related genes including HIF-1alpha are also upregulated during hibernation suggesting a role for this transcription factor in mediating adaptive metabolic responses for hibernation [32, 33] useful in intervention potential for AD and diabetes.

 AICAR, (Aminoimidazole-4-carboxamide-1-*β*-4-ribofuranoside) an agonist of AMPK, is a mimetic of exercise that upregulates pathways common to exercise including the key PGC-1 energy regulator [40]. In theory, and in experimental studies, AICAR intervenes in acute ischemic stress, by activation of protective pathways that are anti-inflammatory, anti-oxidative stress, and prosurvival pathways that promote intervention in ischemic stress pathways induced by exercise. Indeed, in our recent studies, AICAR pretreatment and posttreatment significantly increases tolerance and survival to a severe hemorrhage model of ischemic stress [41].

 AICAR is a very promising candidate for pretreatment of early and late AD since evidence shows that AICAR treatment increased PGC-1alpha as a mimetic of stress. Increases PGC-1 levels dramatically protect neural cells in culture from oxidative-stressor-mediated death and making PGC-1 a target molecule for therapeutic manipulation oxidative stress [42] and candidate target molecule in AD therapy. AICAR intervenes in LPS/A beta-induced inflammatory processes by blocking the expression of proinflammatory cytokines, inhibits reactive oxygen species in astroglial cells, and promotes NGF-induced neurite growth in PC-12 cells [43]. PGC-1 is identified as a target molecule for diabetes as well [44].

 Related studies use nutritional supplements to increase heat shock proteins and key metabolic regulators. Acetyl-L-carnitine induces upregulation of heat shock proteins and protects cortical neurons against amyloid-beta peptide 1–42 mediated toxicity and, thus, is nutritional candidate for intervention in AD [12, 45, 46]. Resveratrol, as well, is among the potential supplement s for AD via manipulation targeting activation of the Sirt-/PGC-1 neuroprotective axis [19, 47]. Other supplements and cocktails are recommended in other studies and reviews.

 Mimetics of environmental stress, in the seemingly unrelated phenomena, hibernation, exercise, heat attack, stroke, severe hemorrhagic stroke trauma, metabolic diseases, and neurological disorders and AD, share common denominators, ischemic, metabolic, protein misfolding, and oxidative stress. The induction of protective pathways that promote survival instead of apoptosis and cell death, associated with energy deficits and inflammatory processes share drug benefits despite the disparity in the acute and chronic disease states. Diabetes drugs then may have potential for AD therapy.

## 6. Telomeres, Aging, and Alzheimer's Disease

 Telomerase is a ribonucleoprotein polymerase that maintains telomere ends by the addition of the telomere repeat, TTAGGG, that declines with age. Telomerase and telomeres are the subject of thousands of current studies and reviews that link telomeres to aging and cancer. It is clear that telomerase function is not restricted only to repair of lost telomere length with age and may interact with the polycomb complex, that impacts various biological processes, including differentiation, maintenance of cell identity, cell proliferation, and stem-cell plasticity. Decline in telomere function links mitochondria, stem cells, and metabolic compromise [48, 49].

 Genetically engineered telomerase-deficient mice are a model system that shows in vivo wide-spread endogenous DNA damage, tissue atrophy, stem-cell depletion, organ system failure and impaired tissue response that mimic age-related changes. The reversal of tissue degeneration in aged telomerase-deficient mice by genetically engineered inducible telomerase activation shows unprecedented evidence for global regeneration of organ systems [50]. Telomerase reactivation in late generation TERT-ER mice rejuvenates mice. The telomerase induction extends telomeres, reduces DNA damage, associated cellular checkpoint responses, restores proliferation in quiescent cultures, eliminates degenerative phenotypes across multiple organs, reverses neurodegeneration, and restores the innate behavioral olfactory avoidance responses [50]. Evidence, that such regeneration occurs in normal aging with activation of telomerase, is, as yet, not available. The role of telomerase and cancer is still unraveling; mimics of UV irradiation, telomere oligonucleotides, induce apoptosis in malignant but not normal cells [16] and offer an anticancer potential for telomere damage response.

 Longer telomere length usually correlates with positive survival response and stress resistance [51]. However telomere *shortening* reduces amyloid brain pathology in mice [52] and mole rats, a rodent model of successful aging, does not show age-related disease vulnerability, and has short telomeres [53].

 The role of telomeres in aging and disease is of major importance as knowledge of operative mechanisms unravel in normal and disease states. At present, telomerase induction appears as an antiaging and rejuvenation potential that may delay vulnerability to AD; however, it is too premature to predict benefits and adverse side effects of treatment. Activation of stem cell repair is a projected pathway with advantage potential for AD.

## 7. Senescence versus Rejuvenation

 The antithesis of telomerase promotion of cell replication and growth is the p16INK4 locus involved in promotion of senescence. The Ink4a/Arf locus encodes 2 tumor suppressor molecules, p16INK4a and Arf, considered the principal mediators of cellular senescence [54, 55]. Expression of p16INK4a and Arf markedly increases in almost all rodent tissues with advancing age, while there is little or no change in the expression of other related cell cycle inhibitors. The age-related increase in Ink4a/Arf expression can be independently attributed to the expression of Ets-1, a known p16INK4a transcriptional activator, as well as unknown Ink4a/Arf coregulatory molecules [54, 55]. Genetic data have firmly established that both p16INK4a and ARF proteins possess significant in vivo tumor suppressor activity. The anti-cancer growth inhibitory activity of the p16INK4a and ARF locus that can arrest cell growth benefit unfortunately can arrest cell growth in cells possessing self-renewal potential like tissue stem cells with a resulting decline the regenerative capabilities of the organ maintained by that stem cell. Decline of this stem cell reserve is a cardinal feature of mammalian aging marking the expression of the INK4a/ARF locus, not only to be a major suppressor of cancer, but also an effecter of mammalian aging [54]. Mimetics that tips the balance between INKA and telomerase, without cancer promotion, are candidates for successful aging. There is already evidence that the replicative state of the cell, normal or cancer, can determine response to telomerase induction [16]. The telomerase global potential is, at once, awesome and frightening; evidence is that aside from extension of telomeres, telomerase is a master regulator with potential for regulation of hundreds of genes with unknown immediate or long-term adverse side effects on nondividing brain cells in normal human aging. The telomere long length and telomerase rejuvenation potential, though highly correlated with longevity, can be independent, as found in the mole rat short telomeres with long life.

## 8. Successful Aging Model

 In contrast with the multiple mouse models of disease and age-accelerated systems, the naked mole rat, living in burrows in arid and semiarid burrows in Africa, represents a model of successful aging. The mole rats are the longest-living rodents known, with a maximum lifespan of 30 years, at least 5 times or longer than expected on the basis of body size [53]. For at least 80% of their life, mole rats maintain normal activity, body composition, reproductive and physiological functions with no obvious age-related increases in morbidity or mortality rate, and cancer resistance. Surprisingly, the mole rats have high levels of oxidative stress and relatively short telomeres, yet they are extremely resilient when subjected to cellular stressors and appear capable of sustaining both their genomic and protein integrity under hostile conditions [56]. The resistance of mole rats to oxidative stress suggests resistance neurological damage. Hypoxic stress by nutrient oxygen deprivation in hippocampal slices of naked mole rat shows that neural tissue is resistant to nutrient oxygen deprivation [56] and likely resistance to AD toxicity. Neuregulin-1 (NRG-1) signaling, critical for normal brain function during both development and adulthood, is sustained throughout development and adulthood in mole rat [57]. Moreover, mean lifespan strongly correlated with levels of NRG-1, and its receptor, linking lifespan and NRG-1 levels. Neuregulin becomes a candidate target molecule for modulation, and the mole rat, a model organism for AD research.

## 9. Immunological Therapy and Innate Immunity

 The major focus of Alzheimer's research is the attractive immunological therapeutic intervention approach to AD. Over 25,000 articles report the progress and perils of immunotherapy in treatment of AD as the focus of pharmaceutical drug discovery. Like induction of stress response to combat the disease challenge, induction of the immune response activates defenses to intervene in AD. Multiple reviews are available on the topic, and only a brief description of this valuable therapeutic approach is included here. An immunological solution has proven to be elusive, complex, costly, and ineffective so far, as the studies of the last decade reveal. Lessons learned include the discovery that although immunization of Amyloid *β* (A*β*) peptide could protect and reverse amyloid pathology in animal models, in human trials, although immunotherapy did clear amyloid plaques, the clearance did not show a cognitive benefit effect in AD patients [58]. The amyloid hypothesis, as a target for AD immunotherapy, is at the crossroads [59]. Hope for (A*β*) vaccines remains, since a subset of patients with antibody titers in the active vaccine study, showed signs of cognitive stabilization [60]. Adverse effects resulted in the discontinuance of human trials in an active vaccine study including meningoencephalitis with AN1792, vasogenic edema, and microhemorrhages with bapineuzumab, and uncertain results of cognitive benefit using passive (A*β*) immunotherapy in a genetic subgroup carriers of the APOE 4 gene [61]. New generation vaccines against (A*β*) peptide, and tau protein, may avoid adverse side effects, and slow progression of cognitive loss. The further refinement of AD DNA epitope vaccines is another immunological approach with promise for clinical trials administered preferably in preclinical stage individuals identified by validated AD biomarkers [62]. Unfortunately, agreement on the underlying cause(s) of AD is not established nor is the optimal immunological target(s).

 An alternative immune therapy approach is the activation of innate immune function conserved throughout evolution, present in ancient organisms, and inducible in humans that does not require the knowledge of the causative agent of AD; rather activates a generalized resistance state. The preserved ancient immune T-cell immunoregulator, the CDR1 peptide of sharks, elicits an immune response in higher organisms and humans. The CDR1 peptide is involved in homeostasis, immunoregulation, response to infection, and reversal of the negative effects of immunosenescence on normal TH1 and TH2 T-cell subsets [63]. The TCR peptide itself restores balance between TH1 and TH2 and stimulates cells remodeling defective heart tissue implicating a role for immune system in cardiac repair [64]. The reversal of immunosenescence may directly impact the vulnerability of elderly to AD, or even provide repair after AD onset.

 Another ancient immune factor is “the unmethylated CpG motifs,” found to be prevalent in bacterial but not vertebrate genomic DNAs [65]. Oligodeoxynucleotides containing CpG motifs activate host defense mechanisms leading to innate and acquired immune responses. The recognition of CpG motifs requires the toll-like receptor. CpG-induced activation of innate immunity protects against lethal challenge from a wide variety of pathogens and has therapeutic activity in murine models of cancer and allergy. CpG motifs also enhance the development of acquired immune responses for prophylactic and therapeutic vaccination [65] and may boost immune function in AD vaccinations.

## 10. Stress Response Activation: Timing and Delivery

 The optimal timing of intervention with alternative trigger induction strategies, intuitively, is prior to the onset of disease in known vulnerable candidates (early onset genetic predisposition, the elderly, prior history of brain damage), or in the early phases when there is detection of AD biomarkers, as is the preferred treatment population for all AD interventions. It is easier to prevent damage, rather than repair damage. However, there is promise for intervention in later stages of AD for delay of progression, or even reversal using triggers of stress resistance by upregulation of tolerance and rejuvenation after damage has occurred in other disease models, as outlined in the references presented above, for use in all stages of AD progression to delay progression or even reverse symptoms.

 In theory, the delivery of mimetics of stress resistance triggers may be by oral, venous injection, or intranasal, since these delivery modes have been used to activate protective and rejuvenation response in rodent models, and in some cases to treat inflammatory human diseases. Especially promising is the use of intranasal delivery in neurodegenerative diseases and stroke [66]. Direct access to the damage brain tissue is attractive and may avoid other potential adverse effects by system-wide treatment.

 From the above discussions, the theoretical benefits of the stress response triggers after disease onset include (1) the upregulation of protective mechanism to restore protein structure, using the inducers of chaperone proteins HSP's; (2) reduction of the increased inflammatory response to the disease states, and oxidative damage cascades, using hibernation like opioid mimetics, innate immune triggers, and endogenous antioxidant element triggers, to protect against further damage; (3) restoration of metabolic homeostasis, proteostasis, and antioxidant protection with the exercise mimetic, AICAR [43]. Cognitive function requires rejuvenation and repair, reduction of cell death, and induction of Nerve Growth Factors found in cells after AICAR treatment.

 Theoretically, the vision is that induction of stress resistance will delay or stop the progression of the disease and even restore cognitive function. More than one trigger may be required, and/or the strategy of induction of stress resistance may be a valuable addition in genetic subpopulations resistant to immune therapies. Lessons learned from history teach us that theory and practice do not always coincide. Until appropriate controlled human clinical trials are explored and analyzed, the actual benefit of the proposed strategy remains unknown.

## 11. Conclusion

 Mimetics of chemical and environmental stress can provide valuable activation of protective pathways with potential in intervention in the pathologies of AD now. The advantages of the activation of stress resistance as alternative strategy include availability, without new drug development, and, in some cases, triggers are already in human use to treat other metabolic, ischemic, and inflammatory disease conditions and pathologies. There is real hope for multiple options in AD intervention drugs presently in testing, alone or in combination with other therapies, especially in genetic subpopulations resistant to immunotherapy or other approaches.
